# Recent advances in understanding bile duct remodeling and fibrosis

**DOI:** 10.12688/f1000research.14578.1

**Published:** 2018-07-31

**Authors:** Marinda Scrushy, April O'Brien, Shannon Glaser

**Affiliations:** 1 Texas A&M University College of Medicine, Temple, TX, USA; 2 Central Texas Veterans Research Center, Temple, TX, USA; 3 Digestive Diseases Research Center, Baylor Scott & White Health, Temple, TX, USA

**Keywords:** cholestasis, cholangiocytes, ductular reaction, bile duct

## Abstract

Cholestatic liver disease encompasses a detrimental group of diseases that are non-discriminatory in nature. These diseases occur over every age range from infancy (biliary atresia) to geriatrics (hepatitis). They also cover both genders in the form of primary sclerosing cholangitis in men and primary biliary cholangitis in women. Oftentimes, owing to the disease progression and extensive scarring, the treatment of last resort becomes a liver transplant. In this review, we will briefly discuss and explore new avenues of understanding in the progression of cholestatic liver disease and possible therapeutic targets for intervention. The greater our understanding into the idiopathic nature of cholestatic liver disease, the better our chances of discovering treatment options to halt or reverse the progression, reducing or eliminating the need for expensive and risky transplants.

## Introduction

The hepatobiliary system, a crucial part of the digestive system, is made up of the liver, gall bladder, and bile ducts, a complex network of tubular intra- and extra-hepatic ducts that converge to drain bile into the duodenum to aid in fat digestion and the transportation of wastes ^1^. The bile ducts are lined by a subset of liver epithelial cells termed cholangiocytes. Cholangiocytes are responsible for the modification of bile fluidity and alkalinity after secretion of the major bile osmolytes and various constituents by the hepatocyte ^1– 3^. These ducts, or channels, are part of a larger three-dimensional structure known as the biliary tree, which branches into the surrounding liver parenchyma ^2^. There is a wide array of disease states specifically targeting the cholangiocytes, termed cholangiopathies, many of which are induced by cholestasis. Cholestasis is defined as a bile flow decrease caused by either defective hepatocyte secretions or the physical obstruction of bile through the intra- and extra-hepatic bile ducts ^4^.

Chronic cholestasis causes destruction of the bile ducts, strictures, and fibrosis. Chronic fibrosis and inflammation can progress to permanent scarring leading to hepatic failure, necessitating the need for a liver transplant. Mouse models of cholestasis include the mechanical model of bile duct ligation (BDL), as well as the 3,5-diethoxycarbonyl-1,4-dihydrocollidine (DDC) diet and the Mdr2 or abcb4 knockout mouse. In the BDL mouse model, a surgical incision is made in the abdomen, and after careful manipulation of the liver, the bile duct is exposed and isolated from the adjoining hepatic artery and portal vein, allowing it to be tied off by surgical sutures. The incision is then closed and cholestasis is allowed to develop ^5^. As a consequence of BDL, adaptive remodeling of bile ducts as well as an increase in ductular tissue density has been observed ^2, 6^. The DDC model was initially used to reproduce steatohepatitis but also has been shown to reproduce conditions observed in chronic cholestatic diseases such as ductular proliferation as well as ductal and liver fibrosis ^7^. Alternatively, the Mdr2 abc4 ^−/−^ knockout mice mimic cholestasis by inducing a deficit in canalicular phospholipid flippase leading to the absence of phospholipids in bile. This causes bile leakage that has been shown to lead to bile duct and portal fibrosis ^8^.

This response is coined the “ductular reaction” and takes place after a cholestatic-inducing injury causes the normally quiescent cholangiocytes to take on a reactive phenotype. This involves an epithelial-to-mesenchymal transition (EMT), cholangiocyte proliferation, and a change to a neuroendocrine phenotype where cholangiocytes begin to secrete and respond to a number of peptides and hormones. This reactive phenotype brings about changes in the extra-cellular matrix (ECM) that leads to the collapse of the bile duct architecture with eventual fibrosis and strictures ^9^. It is hypothesized that the proliferation and other morphologic changes observed after BDL are the liver’s attempt to increase both the surface area and function of cholangiocytes ^6^. The aim of this review is to summarize current research on the cholangiocyte’s response to cholestatic injury, focusing on the role of proliferation as well as autocrine and paracrine signaling molecules and what role these play in the overall change in bile duct morphology.

## Discussion of recent literature

The remodeling of the bile ducts in response to injury results in an increase in elongation, diameter, and branching of the ducts as well as increased intra-ductal mass ^2, 6^. As cholestasis is defined as the buildup of bile in the ductular system, it can be hypothesized that this remodeling serves to find alternative routes to release the bile. This would function not only to reduce its toxic effects on resident cholangiocytes but also to continue its contribution to overall organismal homeostasis through the breakdown of fats for digestion ^4, 6, 10^. Using three-dimensional confocal imaging and reconstruction techniques, Vartak *et al*. described the bile duct changes they saw in BDL mice, demonstrating that the biliary tree transforms by increasing proliferation, branching, and corrugation. These changes led to an overall increase in surface area ^6^. This increase in surface area is significant as cholangiocytes function via secretion and re-absorption of electrolytes to form and modify bile, a mechanism that requires significant surface area ^1, 3^.

One of the key mechanisms involved in bile duct remodeling is cholangiocyte proliferation. As cholangiocytes are not normally mitotically active, this switch is seen as a response to insult ^11^. Numerous studies have outlined that various physiologic chemicals, including gastrointestinal hormones, angiogenic factors, sex hormones, neuropeptides and neurotransmitters, cytokines, and growth factors, play a role in initiating or attenuating biliary proliferation ^12, 13^. One of the main mechanisms by which proliferation is increased or attenuated in response to these chemicals involves the modulation of cAMP levels, as large cholangiocytes proliferate through a cAMP-dependent mechanism ^12^.

Another possible key role of proliferation is that encroaching ductules deliver hepatic progenitors to the liver parenchyma in order to facilitate hepatocyte regeneration ^2^. These liver progenitor cells express both hepatic and biliary markers and have been shown to be involved in hepatic repair after chronic liver injury. Many growth factors and cytokines, including fibroblast growth factor 7 (FGF7), tumor necrosis factor-like weak induce of apoptosis (TWEAK), hepatocyte growth factor, and the Wnt/B-catenin pathway, have been shown to regulate liver progenitor cell function ^14– 17^.

FGF7 is a fibroblast growth factor that has been shown to attenuate liver markers of cholestasis when overexpressed in the DDC mouse model. Takase *et al*. also demonstrated that it was capable of reversing chronic liver injury induced in the DDC model. They increased FGF7 expression after three weeks of liver injury and again found decreased cholestatic markers ^16^. Both FGF7 and TWEAK induce liver progenitor cell proliferation, and FGF7 plays a role in adaptive remodeling by increasing bile duct density ^2, 15^. The effects of hepatic growth factor are mediated by the tyrosine kinase receptor c-Met. Studies in a c-Met knockout mouse showed that there was an increase in bile acid production leading to increased cholestasis and liver injury. Furthermore, mice lacking c-Met had a decreased proliferative capacity of ductal epithelial cells and thus liver regeneration ^17^. Wnt signaling functions through a B-catenin–dependent pathway and non-B-catenin–dependent pathway. Okabe *et al*. demonstrated that, using the DDC mouse model, Wnt signaling via the B-catenin signaling pathway not only increased biliary proliferation but may play a potential role in hepatocyte-to-biliary trans-differentiation ^14^.

Another increasing interest in the story of ductular remodeling is the hepatocyte-to-cholangiocyte trans-differentiation, or metaplasia. Yanger *et al*. demonstrated through the Notch signaling pathway a transition of hepatocyte to biliary epithelial cells that share morphological, molecular, and structural similarities with their native counterparts ^18^. Additional studies have suggested a role for this metaplasia as a means of escaping injury and expanding the bile ducts while retaining the ability to revert back to their native phenotype. Owing in part to the large percentage of the cellular population comprised by hepatocytes, it has been suggested that this plasticity provides a protective element in times of stress where hepatocytes are the primary targets (that is, hepatitis virus). Furthermore, it has been previously demonstrated that human hepatocytes *in vivo* appear phenotypically plastic following liver injury ^19^.

Recent research has focused on the role that microRNAs (miRNAs) may play in inducing the proliferatory phenotype of the injured cholangiocyte. miRNAs are non-coding RNAs that function to post-transcriptionally silence gene expression by binding to the untranslated region of the mRNA, causing destabilization and the prevention of protein expression ^20^. Research has started to focus on miRNA-21 because of its upregulation in multiple cancer cell types ^21^. miRNA-21 has been found to be upregulated in the livers and bile ducts of BDL mice as well as human primary sclerosing cholangitis (PSC) liver samples. miRNA-21 also promotes proliferation in BDL mice and decreases apoptosis. Additionally, *in vitro* studies have demonstrated that it promotes fibrosis through the activation of hepatic stellate cells via a Smad-7 inhibitory mechanism ^11^.

Changes in ECM proteins, such as integrins, also play a significant role in proliferation. Integrins are a specific type of trans-membrane cellular matrix protein that functions to anchor intra-cellular components to the ECM, attaching to specific cytoskeletal molecules such as laminin and fibronectin ^20, 22^. The structural nature of integrins, when bound, allows for the initiation of signaling cascades that regulate cellular functions such as proliferation and morphological change ^20^. One specific integrin, αvβ6, has been found to be upregulated in injured epithelial cells and plays a role in initiating fibrosis by activating transforming growth factor beta 1 (TGF-β1) ^22^. Connective tissue growth factor (CTGF) is another important ECM component of which cholangiocytes are among the primary sources. CTGF and integrin αvβ6 work together to modulate cellular matrix interactions and activate key players in the ductular reaction and fibrosis ^23^.

The initiation of ductular reactions is characterized by cholangiocyte changes at the molecular level as well. A characteristic of reactive cholangiocytes is a change to a neuroendocrine phenotype as evidenced by their expression of neuroendocrine markers such as chromogranin A and N-CAM ^24^. This phenotypic switch is characterized by increases in both secretion and responsiveness to certain neurohormones and neuropeptides. This induces a critical autocrine and paracrine signaling network that contributes to overall bile duct remodeling by tipping the balance between proliferation and bile duct loss as well as increasing fibrosis ^25^. Recent research has shown that neuropeptide Y (NPY) plays an important role in this process. Cholangiocytes express NPY receptors as well as secrete and respond to NPY during cholestasis with increasing proliferation ^26^. Other neuroendocrine players that upregulate cholangiocyte proliferation include substance P, galanin, gonadotropin-releasing hormone (GnRH), and insulin-like growth factor 1 (IGF1) ^12, 27, 28^. Alternatively, melatonin and serotonin have also been shown to attenuate the ductular response ^24, 29^.

Galanin has recently been shown to be upregulated in the biliary system of mice that have undergone BDL. Furthermore, when galanin was administered to BDL rats, cholangiocyte proliferation was increased because of binding of the galanin receptor 1 with subsequent increases in cAMP ^28^. New research has also outlined a role for IGF1 in the reactive nature of cholangiocytes. Cholangiocytes have previously been shown to express the IGF1 receptor. A recent study demonstrated that in response to increased IGF1 in a rat model of cholestatic liver disease, the cholangiocytes increased both their proliferation and ECM deposition. These upregulated processes led to an increase in fibrosis, a key step in bile duct modulation in cholangiopathies. This ductular reaction may play a role in the activation of surrounding fibroblasts, which also respond with an increase in ECM ^30^.

Another key proliferative player is GnRH, which increases cholangiocyte proliferation ^31^. Additionally, melatonin has been shown to attenuate cholangiocyte proliferation and recent research has investigated how these two neurohormones interact to alter the cholangiocyte response ^25, 29, 32^. Cholangiocytes express GnRH receptors 1 and 2, respectively, and through the action of GnRH, cholangiocyte proliferation has been shown to increase via a cAMP-dependent mechanism. This mechanism takes place primarily through the action of the GnRH receptor 1 ^31^. Melatonin has a regulatory function on GnRH secretion, attenuating its proliferatory effects by decreasing its expression in cholangiocytes ^25^.

An important consequence of chronic cholestasis is bile duct fibrosis. Key mediators of the ductular reaction, such as neuropeptides and neurohormones, that upregulate cholangiocyte proliferation also play a role in upregulating bile duct fibrosis ^33^. Cholangiocytes play a direct key role by activating and secreting pro-fibrotic cytokines. Furthermore, cholangiocytes themselves can produce ECM components, such as collagen I and III ^30^. The EMT is a key event, triggered by injury, where cholangiocytes revert to a mesenchymal phenotype, allowing them to play a key role in fibrosis ^30^. EMT involves functional and morphological changes such as loss of cell-to-cell adhesion properties and apical-basal polarity ^34^. This transition is considered one of the first steps in developing the “reactive” cholangiocyte phenotype. Although cholangiocytes have been shown to express some markers of EMT, this remains an area of controversy as recent lineage-tracing studies have demonstrated no concrete evidence of this change ^35^.

## Conclusions

In summary, research has made great advances in the field of cholestatic liver injury. These advances are a result of both hard work and dedication from the liver research community as a whole with new advances in the understanding of the roles played by the cholangiocyte during injury and repair through subsequent signaling molecules and their mechanisms. These mechanisms, such as EMT, the role of various hormones in the pro-fibrogenic phenotype, and the role of cAMP in proliferation, provide valuable insight and potential therapeutic targets in the fight against liver disease.
